# Aspergillosis in Intensive Care Unit (ICU) patients: epidemiology and economic outcomes

**DOI:** 10.1186/1471-2334-13-29

**Published:** 2013-01-23

**Authors:** John W Baddley, Jennifer M Stephens, Xiang Ji, Xin Gao, Haran T Schlamm, Miriam Tarallo

**Affiliations:** 1 Department of Medicine, Division of Infectious Diseases, University of Alabama at Birmingham, 1900 University Boulevard, 229 Tinsley Harrison Tower, Birmingham, AL, 35294-0006, USA; 2 Pharmerit North America LLC, Bethesda, MD, USA; 3 Pfizer Inc, New York, NY, USA; 4 Pfizer Inc, Rome, Italy

**Keywords:** Aspergillosis, Voriconazole, Fluconazole, ICU, Length of stay, Hospital costs

## Abstract

**Background:**

Few data are available regarding the epidemiology of invasive aspergillosis (IA) in ICU patients. The aim of this study was to examine epidemiology and economic outcomes (length of stay, hospital costs) among ICU patients with IA who lack traditional risk factors for IA, such as cancer, transplants, neutropenia or HIV infection.

**Methods:**

Retrospective cohort study using Premier Inc. Perspective™ US administrative hospital database (2005–2008). Adults with ICU stays and aspergillosis (ICD-9 117.3 plus 484.6) who received initial antifungal therapy (AF) in the ICU were included. Patients with traditional risk factors (cancer, transplant, neutropenia, HIV/AIDS) were excluded. The relationship of antifungal therapy and co-morbidities to economic outcomes were examined using Generalized linear models.

**Results:**

From 6,424 aspergillosis patients in the database, 412 (6.4%) ICU patients with IA were identified. Mean age was 63.9 years and 53% were male. Frequent co-morbidities included steroid use (77%), acute respiratory failure (76%) and acute renal failure (41%). In-hospital mortality was 46%. The most frequently used AF was voriconazole (71% received at least once). Mean length of stay (LOS) was 26.9 days and mean total hospital cost was $76,235. Each 1 day lag before initiating AF therapy was associated with 1.28 days longer hospital stay and 3.5% increase in costs (p < 0.0001 for both).

**Conclusions:**

Invasive aspergillosis in ICU patients is associated with high mortality and hospital costs. Antifungal timing impacts economic outcomes. These findings underscore the importance of timely diagnosis, appropriate treatment, and consideration of *Aspergillus* as a potential etiology in ICU patients.

## Background

Invasive aspergillosis (IA) is an important cause of morbidity and mortality in patients with neutropenia, hematologic malignancy and organ transplants. Despite improvements in diagnostic modalities and the antifungal armamentarium, including broad-spectrum azoles and the echinocandin antifungals, mortality remains high [1-3]. Because of advances in medical care, there has been an expansion in the spectrum of patients at risk for IA beyond the traditional risk groups. An emerging and understudied population is critically ill patients admitted to the intensive care unit (ICU) [4-6]. The extended survival of ICU patients related to improvements in supportive and intensive care, progress in the treatment of respiratory failure, as well as treatment of bacterial infections all may increase the risk of IA in the ICU population [7].

The incidence of IA among ICU patients is estimated to be as high as 7%; however, the frequency of IA is difficult to determine because of several important limitations of IA diagnosis [5]. For example, classic radiographic signs (“halo” or “air crescent”) of IA are not always present in ICU patients, determination of *Aspergillus* colonization versus infection is problematic, and many diagnostic studies have not yet been validated adequately in non-neutropenic patient populations [4,8]. Also lacking among ICU patients with IA are studies evaluating antifungal therapy and relationship to economic outcomes [9-11]. As diagnostic methods for IA improve and patient survival is extended related to improvements in supportive and intensive care, an increased incidence of IA in ICU patients should be expected.

A better understanding of IA, its treatment, and economic outcomes in ICU patients are needed. To date, few cohorts of ICU patients with IA have been evaluated thoroughly [4,5,10,12-14]. Herein, with use of a large administrative database we describe antifungal treatment and clinical and economic outcomes of ICU patients with aspergillosis.

## Methods

### Setting and design

This is a retrospective, observational analysis of multi-center ICU patient data evaluating clinical and economic outcomes of patients with invasive aspergillosis.

### Data collection

Data were derived from Premier’s Perspective™ Comparative Database, a hospital-based, service-level database that provides detailed patient-level resource utilization, diagnoses and procedures from a nationally representative group of > 600 US hospitals [11,15]. Inpatient data from the years 2005 through 2008 were abstracted and included patient demographics, principal and secondary diagnoses and procedures codes (according to the International Classification of Disease 9th revision, ICD-9), hospital and ICU length of stay (LOS), cost and charge details, and mortality and discharge status.

### Study population

**Figure 1:**
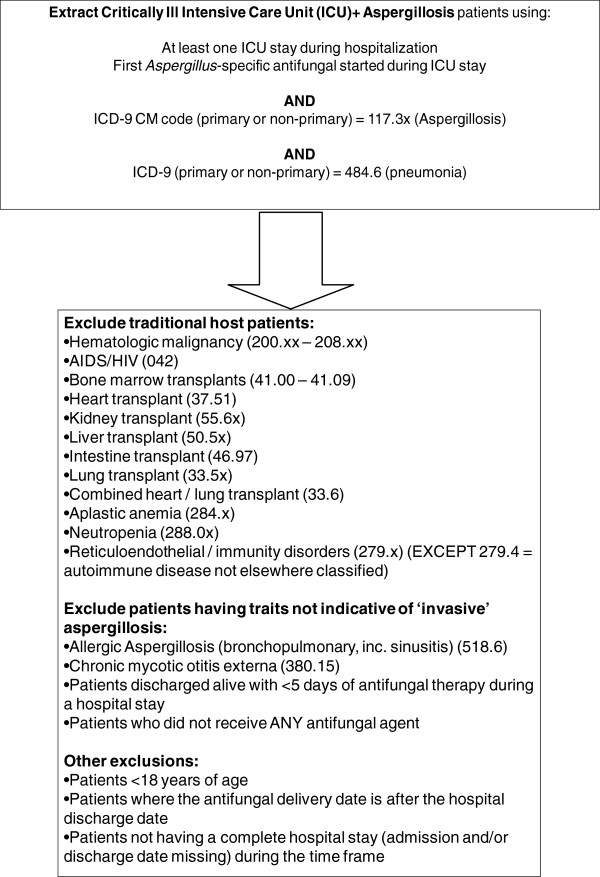
Patient selection schematic.

We included ICU patients with a diagnosis of invasive aspergillosis who started antifungal therapy during their ICU stay and received at least 5 days of antifungal therapy. A case of invasive aspergillosis was defined as a patient at least 18 years-of-age who received a primary or secondary diagnosis code of “aspergillosis” (ICD-9 code 117.3x) plus “pneumonia in aspergillosis” (ICD-9 code 484.6) [16]. In order to focus only on the emerging population of ICU patients, the following patient populations with “traditional” risk factors for IA were excluded: Hematologic malignancy (200.xx – 208.xx); AIDS/HIV (042); bone marrow transplants (41.00 – 41.09); heart transplant (37.51); kidney transplant (55.6x); liver transplant (50.5x); intestine transplant (46.97); lung transplant (33.5x); combined heart / lung transplant (33.6); aplastic anemia (284.x); neutropenia (288.0x); reticuloendothelial / immunity disorders (279.x, except 279.4 , autoimmune disease not elsewhere classified). Patients having non-invasive asperillosis were also excluded: allergic aspergillosis (sinusitis) (518.6); chronic mycotic otitis externa (380.15). For patient comorbidity, the Charlson Comorbidity Index (CCI) was calculated based on Deyo’s method using ICD-9 CM diagnosis codes [17,18]. The Schematic for patient selection is demonstrated in Figure 1.

### Antifungal therapy

Standardized charge codes, in billing records, were used to identify the following antifungal drugs that were administered during hospitalization: voriconazole, caspofungin, micafungin, anidulafungin, itraconazole, fluconazole, posaconazole, and amphotericin B. The first use of an antifungal drug was identified as the *‘Index event’* and reflected days from hospital admission. The length of therapy (days) was computed for each patient. Antifungal therapies were further categorized and labeled as “*monotherapy*” (defined as patients starting with only one antifungal therapy within 3 days of the initial antifungal treatment) and “*combination therapy*” (defined as patients starting with more than one antifungal therapy within 3 days of the initial antifungal treatment).

### Outcomes

The primary outcome, length of stay (LOS), for each hospitalization was abstracted from the dataset, whereas length of ICU stay and Mechanical Ventilation (MV) were computed using the standardized charge codes within billing records. Total patient costs were based on the actual hospital cost to treat the patient and were provided with the dataset. Costs incurred among different hospital departments were also generated using billing records.

### Statistical analyses

Descriptive statistics were calculated for demographic and clinical characteristics as well as for antifungal therapies and resource utilization. To further assess the variation in resource use among patients, multiple regression analyses were performed to characterize the association of independent variables with (1) hospital LOS (defined as time to discharge alive) and (2) hospital cost among survivors and non-survivors. The term *cost* for the purposes of this analysis refers to actual costs incurred by the provider or hospital to administer treatment and patient care; potential patient copayments were not included. To avoid competing risks (impact of mortality on LOS), only patients who survived at least 30 days were included in the regression analyses. Given that LOS and cost data were not normally distributed, generalized linear models (GLMs) were used with Gamma distributions. Variables significant at α = 0.05 level in the univariate analyses (Pearson correlations) and/or variables clinically relevant were included in multivariable analyses. Independent variables in the primary models included initial antifungal therapy (excluding fluconazole), mechanical ventilation, acute renal failure diagnosis, hospital type (teaching vs nonteaching hospital), insurance status, hospital location, severity of illness (using APR-severity, extreme vs. major, and timing of antifungal therapy (i.e., AF index event).

For the primary model, timing of antifungal therapy was a continuous variable, defined as number of days from date of hospital admission to the start date of initial AF therapy. To better ascertain delay in appropriate antifungal therapy, a second model was constructed that included fluconazole (versus other antifungal agents) in addition to the variables above. For this model, timing of antifungal therapy was defined based on the receipt on aspergillus-active therapy. Here, a “delay” in therapy meant that patients received initial therapy with fluconazole and then were switched to an aspergillus-active therapy. Statistical analyses were performed using SAS, version 9.2 (SAS Institute). All statistical tests were two-sided and performed using a 5% significance level (α = 0.05).

## Results

**Figure 2:**
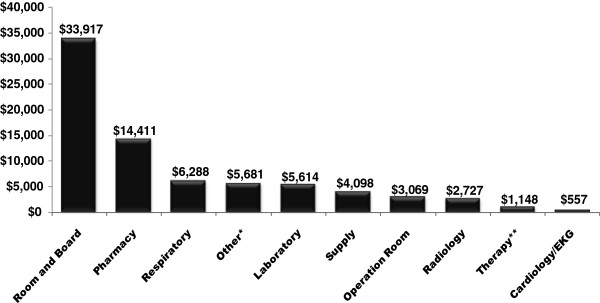
Hospital Costs for ICU Patients with Aspergillosis (N = 412).

Of 6,424 unique patient hospitalizations with aspergillosis abstracted from the dataset, after exclusion criteria were applied, 412 (6.4%) ICU patients with IA were identified and analyzed. The complete ICU population during the four-year study period was 2,470,118 patients, resulting in an IA prevalence of 0.017%. Patient characteristics and outcomes are listed in Tables 1 and 2. Mean age at ICU admission was 63.9 years; 52.9% were male and 67.7% white. Frequent underlying conditions were acute respiratory failure (76.0%), acute renal failure (41.3%), chronic obstructive pulmonary disease (36.9%) and septicemia/septic shock (35.9%). Two-hundred ninety-eight (72.3%) patients received mechanical ventilation at least one day. Mean hospital LOS was 26.9 days and mean length of ICU stay was 15.8 days. In-hospital mortality was 45.6%, with 33.1% dead before 30 days of hospitalization. Average total cost per patient was $76,235, and the majority of costs were related to room and board (Figure 2).

**Table 1:** Patient characteristics

**Characteristic**	**N = 412 (%)**
Mean Age (±SD)	63.9 (14.4)
Male Gender	218 (52.9)
White Race	279 (67.7)
Urban Hospital Location	383 (93.0)
Teaching Hospital	207 (50.2)
Geographic region	
Northeast	56 (13.6)
South	170 (41.3)
Midwest	113(27.4)
West	73 (17.7)
**Comorbidities (ICD-9)**^**1**^	
Acute Respiratory Failure (518.81, 518.84)	313 (76.0)
Mechanical Ventilation	298 (72.3)
Acute Renal Failure (584.9, 584.5)	170 (41.3)
COPD (491.21, 496)	152 (36.9)
Septicemia or Septic Shock (038.9, 785.52)	148 (35.9)
CHF (428.0)	122 (29.6)
Hypertension (401.9)	106 (25.7)
Anemia (285.9)	96 (23.3)
Thrombocytopenia (287.4, 287.5)	83 (20.5)
Acute Steroids	315 (76.5)
Dialysis	72 (17.5)
Charlson Comorbidity index: Mean (SD); (range)	2.5 (2.0); (0.0 – 10.0)

^1^Defined by ICD-9 codes, except for steroid use and dialysis. Steroid use included the use of corticosteroids such as dexamethasone, methylprednisolone, prednisolone, and prednisone during the hospital stay. *SD*, standard deviation; *COPD*, chronic obstructive pulmonary disease; *CHF*, congestive heart failure.

**Table 2:** Outcomes and resource utilization

**Patient characteristics and outcomes**	**ICU + IA patients (N = 412)**
Length of stay (days), Mean (SD); *(min – max)*	26.9 (25.0); (1.0 – 269.0)
Survivors length of stay, Mean (SD); *(min – max)*	29.2 (28.2); (4.0 – 269.0)
Non-survivors length of stay, Mean (SD); *(min – max)*	24.2 (20.4); (1.0 – 121.0)
Hospital ICU stay (days), Mean (SD); *(min – max)*	15.8 (15.3); (1 – 114.0)
Re-hospitalization ^1^, n (%)	5 (1.21%)
Mortality within hospital, n (%)	188 (45.63%)
Expired within 30 days of hospitalization, n (%)	117 (33.05%)
No. of patients with at least one day on mechanical ventilation, n (%)	298 (72.33%)
Length of mechanical ventilation (days), Mean (SD); *(min – max)*	16.5 (15.8); (1.0 – 111.0)
No. of patients with bronchoscopy (%)	60 (14.56%)
No. of patients with needle biopsy (%)	221 (53.64%)
**Antifungal Therapies**	
Index Event (IE) Day ^2^ , Mean (SD); *(min – max)*	8.6 (9.2); (1.0 – 85.0)
No. of Antifungal therapies per patient, Mean (SD); *(min – max)*	1.70 (0.81); (1.00 – 4.00)
Length of Antifungal therapy (days), Mean (SD); *(min – max)*	14.6 (13.0); (1.0 – 122.0)
Length of therapy (Survivors)	16.3 (12.8); (5.0 – 122.0)
Length of therapy (Non-survivors)	12.6 (13.1); (1.0 – 93.0)

^1^ Re-hospitalization to the same hospital, within 4 months of discharge.

^2^ Index Event Day = Inpatient day when the antifungal therapy was initiated.

*ICU*, intensive care unit; *IA*, invasive aspergillosis.

### Antifungal therapy

On average, antifungal therapy was initiated on day 9 of the hospital stay, with a mean length of therapy 14.6 days while in the hospital (Table 2). The mean number of antifungal drugs received per patient was 1.7 (range, 1–4). Most patients (85.9%) received monotherapy. Overall, 292 (70.9%) patients received at least one dose of voriconazole; 161 (39.1%) caspofungin; 53 (12.9%) amphotericin B; 34 (8.3%) itraconazole and 22 (5.4%) micafungin or anidulafungin. Combination therapy was used in 58 (14.1%) of 412 patients and the most frequent combination was voriconazole plus caspofungin. Ninety-eight (27.7%) patients received fluconazole before the addition or change to a drug with *Aspergillus* activity. A summary of specific antifungal therapy can be found in Table 3.

**Table 3:** Antifungal therapy (N = 412 patients)

**Description**	**VORI**	**CASPO**	**FLUC**	**ITRA**	**AMB**	**POSA**	**MICA**	**ANID**
Used drug at least once (%)	292 (70.87)	161 (39.08)	117 (28.40)	34 (8.25)	53 (12.86)	3 (0.73)	13 (3.16)	9 (2.18)
Used as initial monotherapy (%) (N = 354)	166 (46.89)	63 (17.80)	98 (27.68)	12 (3.39)	12 (3.39)	0 (0.00)	2 (0.56)	1 (0.28)
Used as initial combination therapy (%) (N = 58)	53 (91.38)	38 (65.51)	4 (6.90)	10 (12.50)	9 (11.25)	3 (5.08)	5 (8.62)	5 (8.62)
Length of initial therapy, days (SD)	12.1 (12.3)	9.1 (8.4)	6.0 (4.7)	8.4 (11.3)	7.3 (8.3)	14.3 (18.0)	7.7 (6.6)	10.6 (10.3)
Length of initial therapy (Survivors)	12.8 (12.6)	10.9 (7.7)	6.7 (5.0)	9.4 (12.6)	7.1 (6.9)	14.3 (18.0)	8.7 (7.8)	11.3 (10.8)
Length of initial therapy (Non-survivors)	11.2 (12.0)	7.2 (8.8)	6.4 (4.5)	6.1 (7.2)	7.4 (9.1)	-	6.5 (5.2)	5.0 (−)
Mortality (% who received drug at least once)	125 (42.81)	79 (49.07)	66 (48.89)	10 (29.41)	33 (62.26)	0 (0.00)	6 (46.15)	1 (11.11)
Hospital admission to discharge, days (SD)	27.8 (26.3)	31.1 (31.1)	33.9 (24.2)	28.9 (19.5)	30.7 (25.2)	31.0 (21.2)	25.3 (17.0)	32.8 (20.7)
Drug initiation to discharge, days (SD)	19.2 (21.7)	21.0 (26.1)	25.1 (22.4)	21.7 (16.3)	23.2 (23.2)	24.0 (18.0)	20.5 (18.4)	26.3 (15.3)

*VORI*, voriconazole; *CASPO*, caspofungin; *FLUC*, fluconazole; *ITRA*, itraconazole; *POSA*, posaconazole; *MICA*, micafungin; *ANID*, anidulafungin.

SD=standard deviation.

### Factors associated with hospital LOS or cost

The multivariable regression analyses of factors associated with LOS or cost can be found in Table 4. For the primary model (excluding fluconazole) the only independent predictor of LOS identified was timing of initial antifungal therapy: a one day delay in starting anti-aspergillus antifungal therapy was associated with approximately 1.28 days longer LOS (p < 0.0001). With regard to hospital cost, patients who received mechanical ventilation or who had acute renal failure incurred 85.4% and 43.7% more costs, respectively, when compared to those who did not (p < 0.001 for both). Severity of illness (extreme versus major) using the APR measure, was associated with a 57% increase in hospital costs (p < 0.05). The timing of initial antifungal therapy also had a significant impact on hospital cost: a one day delay in starting anti-aspergillus antifungal therapy was associated with approximately 4% higher total costs per day (p < 0.0001).

**Table 4:** Multivariable analyses of factors associated with hospital length of stay or costs among patients who survived at least 30 days

**Variable**	**Length of stay (LOS) (GLM gamma-identity link, N = 163)**		**Total cost (GLM gamma-log link, N = 162)**	
	**β estimate (days)**	**95% Wald CI for β estimate**	**Exp (β) estimate**	**95% Wald CI for Exp (β) estimate**
Antifungal Therapy				
Other vs. voriconazole	5.33	(−3.43, 14.10)	1.527	(.996, 2.342)
Echinocandins vs. voriconazole	−1.76	(−7.76, 4.23)	.925	(.729, 1.174)
Mechanical Ventilation	7.32	(−.218, 14.87)	1.854****	(1.41, 2.44)
Acute Renal Failure	8.57	(−1.08, 18.23)	1.437**	(1.10, 1.87)
Area				
South vs. West	−3.08	(−14.18, 8.03)	.591*	(.382, .917)
Northeast vs. West	−4.88	(−16.98, 7.23)	.558*	(.358, .872)
Midwest vs. West	−4.27	(−15.81, 7.28)	.588*	(.378, .913)
APR-severity	5.71	(−0.68, 12.09)	1.570*	(1.01, 2.24)
Teaching Hospital	−1.25	(−5.93, 3.43)	1.015	(.829, 1.242)
Payer				
Others vs. Medicare	-.712	(−8.14, 6.71)	.964	(.680, 1.37)
Commercial-Indemnity vs. Medicare	-.814	(−7.84, 6.21)	.860	(.657, 1.12)
Managed Care vs. Medicare	4.34	(−3.04, 11.71)	1.603**	(1.16, 2.22)
Medicaid vs. Medicare	5.69	(−8.06, 19.44)	1.385	(.963, 1.99)
Timing of antifungal therapy^1^	1.28****	(0.89, 1.67)	1.035****	(1.02, 1.05)

*p < 0.05 ** p < 0.01 ***p < 0.001 **** p < 0.0001.

^1^ AF index event (defined as days from hospital admission to initial antifungal start date).

For the sub-analysis of the model including fluconazole there was one notable difference in the results: patients who received initial fluconazole (versus initial voriconazole) on average had almost 6 days longer LOS than patients who received initial voriconazole (p < 0.05) and 33% increase in hospital costs (p < 0.05).

## Discussion

This large US study is one of few to investigate economic outcomes in patients with aspergillosis and provides an understanding of the epidemiology of aspergillosis among ICU patients given newly available antifungal treatment strategies. Our findings, using 2005–2008 multi-center administrative data, demonstrate that ICU patients with aspergillosis have many co-morbidities, high mortality and incur substantial hospital costs. The majority of costs per patient were driven by hospital LOS (room and board).

Risk factors for developing aspergillosis in the ICU population include high-dose corticosteroids and co-morbidities such as renal failure, COPD, and diabetes [4-7]. Among our patient population, 76% of the study cohort received acute high dose corticosteroid therapy during the inpatient stay. Many had respiratory failure; with 72% requiring mechanical ventilation. Other frequent co-morbidities included acute renal failure, SIRS, COPD and septicemia/shock. The frequency of co-morbidities illustrate the broad spectrum of illnesses that may be present in non-traditional IA hosts, and underscores the importance of consideration of aspergillosis in critically ill patients.

Our study reported in-hospital mortality of 46%. Several studies reporting data from 1997–1999 described higher mortality rates (75-80%) in ICU patients with IA [9,19,20]. However, these studies pre-date the availability improved diagnostics and newer antifungal agents studied in our analysis. Our study may reflect a recent trend of decreasing mortality in patients with aspergillosis [11,21,22]. Kim and colleagues describe crude in-hospital mortality of 36.7% reported for years 2005–2006 in a broad national cohort of hospitalized patients with IA [11]. This decreasing trend may have resulted from numerous factors, including antifungal therapy, more timely and accurate diagnostic approaches, earlier initiation of therapy, or technological advances in ICU care [21,22].

The 2008 Infectious Diseases Society of America guidelines for the treatment of aspergillosis recommend voriconazole as primary therapy [23]. Consistent with those guidelines, among our cohort, voriconazole was the most widely used antifungal, with 47% receiving it as first-line monotherapy and 71% of the patients receiving it during hospitalization. Caspofungin was second-most common antifungal with *Aspergillus* activity employed, used initially in 18% of patients. Use of combination antifungal therapy for aspergillosis was not common, administered in only 14% of patients, and is not recommended in recent guidelines [23]. Fluconazole, which does not have *Aspergillus* activity, was used as the initial antifungal in 28% of patients. We were unable to determine from these data if initial fluconazole was appropriate initial therapy (either as prophylaxis or treatment of invasive candidiasis) for these patients. We suspect that empirical treatment or prophylaxis for *Candida* infections may have been attempted in some patients, with a change to aspergillus-active therapy once the diagnosis of aspergillosis was suspected or confirmed.

Our study indicates a potential impact of antifungal timing on economic outcomes. Multiple regression analyses revealed that delay of antifungal therapy resulted in increased LOS and hospital costs: each 1 hospital day lag of initial antifungal therapy was associated with a 1.28 day LOS increase and a 4% cost increase. However, it is important to note that our definition of timing of antifungal therapy was based on hospital admission date to antifungal start date, as the precise timing of diagnosis of IA could not be defined with these data. These findings support the need for better, rapid diagnostic methods for IA in ICU patients and potentially earlier institution of antifungal therapy. A high index of suspicion of IA is warranted in critically ill patients. Length of stay or costs were not impacted by initial voriconazole use (compared to echinocandins or other antifungal use) in our analyses. In contrast, Kim and colleagues found that initial voriconazole, caspofungin, or amphotericin B lipid complex were independently associated with a reduced hospital LOS and costs [11]. An important difference was that Kim and colleagues analyzed all hospitalized patients with aspergillosis, not just ICU patients. Because of the severity of illness and increased mortality among ICU patients, perhaps antifungal therapy, in relation to co-morbidities, may have less of an impact on economic outcomes in critically ill patients. In the sub-analysis, initial fluconazole use (versus initial voriconazole) was associated with increased LOS; however, from these data it is unclear if this represents initial treatment of candidiasis or a true “delay” in appropriate antifungal therapy for IA.

Our study has limitations typical for administrative database analyses that must rely on diagnosis coding. Diagnosis of IA is difficult, and diagnostic guidelines have not yet been written for ICU patients without traditional risk factors. In addition, there are no clearly defined ICD-9 codes to identify “invasive aspergillosis.” As such, misclassification may have occurred in our cohort. Our case diagnosis included code 117.3 (aspergillosis) plus the 484.6 code (pneumonia with aspergillosis). In addition, we excluded patients with non-invasive codes such as allergic aspergillosis (sinusitis) 518.6 and chronic mycotic otitis externa. While the database did not provide clinical information about laboratory test results, billing data revealed that approximately two-thirds of the patients had a bronchoscopy, needle biopsy, or *Aspergillus* serology, supporting the diagnosis of IA. Nevertheless, as a recent validation study reported, specificity using ICD-9 codes for aspergillosis ranges from 28-88% [16].

Another limitation is the competing risk issue coming from the high mortality risk in this ICU population, which usually complicates the models constructed to evaluate LOS. We attempted to reduce this confounding effect by examining the outcomes separately (discharge alive and discharge dead) and redefining the non-survivors as patients who expired within 30 days of hospitalization to minimize variance between survivors and non-survivors. Given that the primary interest of this study was not to identify predicting factors of ICU mortality, rather to examine the impact of antifungal therapy on the outcome of LOS/discharge alive, the problem of competing risk should be less relevant in the GLM analyses among re-defined survivors [24,25].

A sensitivity analysis (not shown) was performed with use of Cox regression analysis and resulted in similar findings. Additional limitations include the study’s retrospective, nonrandomized design; however, the use of observational data adds a unique and important aspect to comparative effectiveness assessments beyond the clinical trial. Patients’ activity outside of the Premier group of hospitals was not captured including costs charged outside of the hospital billing system such as physicians’ fees; however, they are not likely to contribute significantly to overall costs.

## Conclusion

This large, US retrospective analysis describes the epidemiology of ICU patients with aspergillosis, an emerging non-traditional group. Economic outcomes including LOS and hospital costs are impacted by antifungal therapy and patient co-morbidities. These findings underscore the importance of awareness of potential aspergillosis, timely diagnosis and appropriate, early treatment among ICU patients.

This work was presented in part at the Society of Critical Care Medicine’s 40th Critical Care Conference, January 2011, San Diego, CA.

## Competing interests

JS, XJ and XG are employees of Pharmerit North America LLC and served as paid consultants on behalf of Pfizer, Inc., to perform the analyses. Pharmerit North America LLC purchased the data source, Premier’s Perspective™ Database, on behalf of the sponsor, Pfizer, Inc. Permission of use of the data source was granted per a standard licensing contract with Pharmerit North America, LLC. JB has received honoraria for advisory board work for Merck and Mayne Pharma. JB served as a paid consultant for Pfizer, Inc., for study concept and design. JB made the decision to submit the study for publication and did not receive funding for manuscript preparation or interpretation of results. Miriam Tarallo and Haran Schlamm are full-time employees of Pfizer, Inc.

## Authors’ contributions

JWB conceived of the study, participated in the design of the study, review and interpretation of results, and drafted the manuscript. JMS developed the study protocol and analysis plan, reviewed and interpreted results, and assisted in drafting the manuscript. XJ participated in the development of the statistical analysis plan, performed the statistical analyses, assisted in drafting the manuscript, and in coordination of the study. XG developed the study protocol and analysis plan, conducted statistical analysis, reviewed and interpreted results, and conducted critical revision of the manuscript for important intellectual content. HTS participated in the design of the study, reviewed and interpreted results, and conducted critical revision of the manuscript for important intellectual content. MT conceived of the study and participated in the design of the study, reviewed and interpreted results, and conducted critical revision of the manuscript for important intellectual content. All authors approved and read the final manuscript.

## Pre-publication history

The pre-publication history for this paper can be accessed here:

http://www.biomedcentral.com/1471-2334/13/29/prepub
